# Supplementary material for: Lactobacillus rhamnosus GG Derived Extracellular Vesicles Modulate Gut Microbiota and Attenuate Inflammatory in DSS-Induced Colitis Mice
Source: Nutrients. 2021 Sep 23;13(10):3319. doi: 10.3390/nu13103319 (PMC8541209; doi:10.3390/nu13103319)

Lactobacillus rhamnosus GG derived extracellular vesicles modulate gut microbiota and attenuate inflammatory in DSS-induced colitis mice

Lingjun Tong[1], Xinyi Zhang[1], Haining Hao[1], Qiqi Liu[1], Zihan Zhou[1], Xi Liang[1], Tongjie Liu[1], Pimin Gong[1], Lanwei Zhang[1], Zhengyuan Zhai[2,] Yanling Hao[2,3], Huaxi Yi[1*]

[1] College of Food Science and Engineering, Ocean University of China, 5 Yushan Road, Qingdao 266003, P. R. China

[2] Key Laboratory of Functional Dairy, Co-constructed by Ministry of Education and Beijing Municipality, College of Food Science and Nutritional Engineering, China Agricultural University, Beijing, China

[3] College of Food Science and Nutritional Engineering, China Agricultural University, 17 Qing Hua East Road, Hai Dian District, Beijing, 100083, China

*Correspondence: yihx@ouc.edu.cn (H.X. Y.); Tel.: +86-0532-8203-2162 (H.X.Y.)

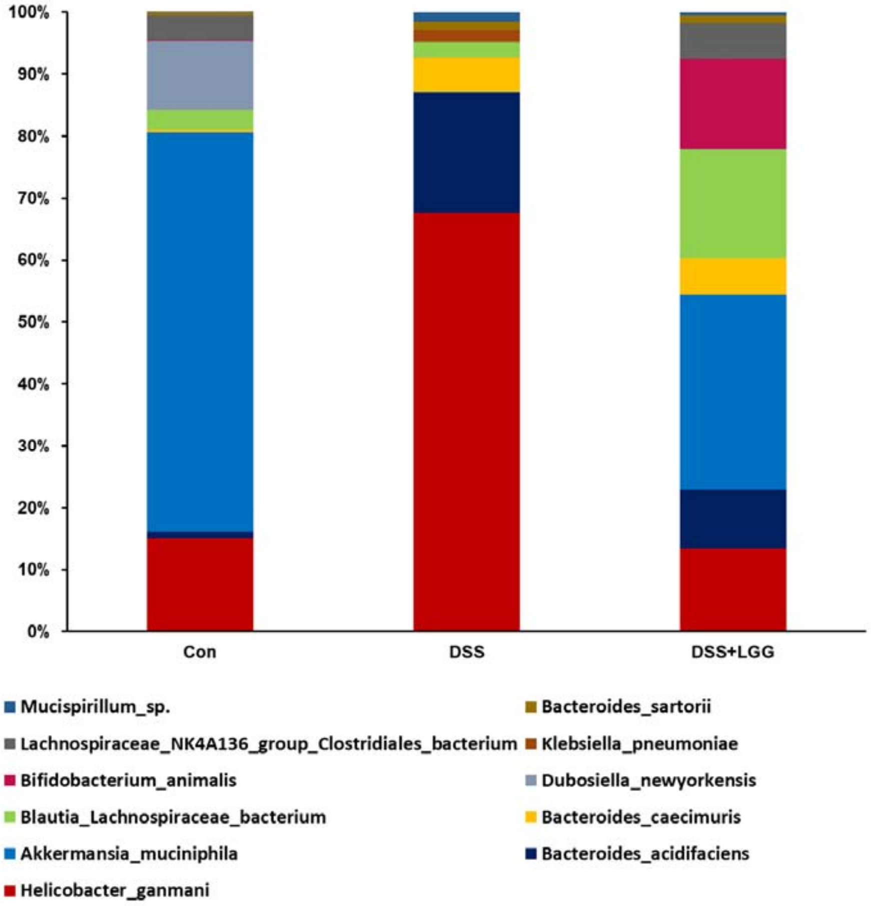

Fig. S1 Bar plots of the taxonomic composition at the species level.

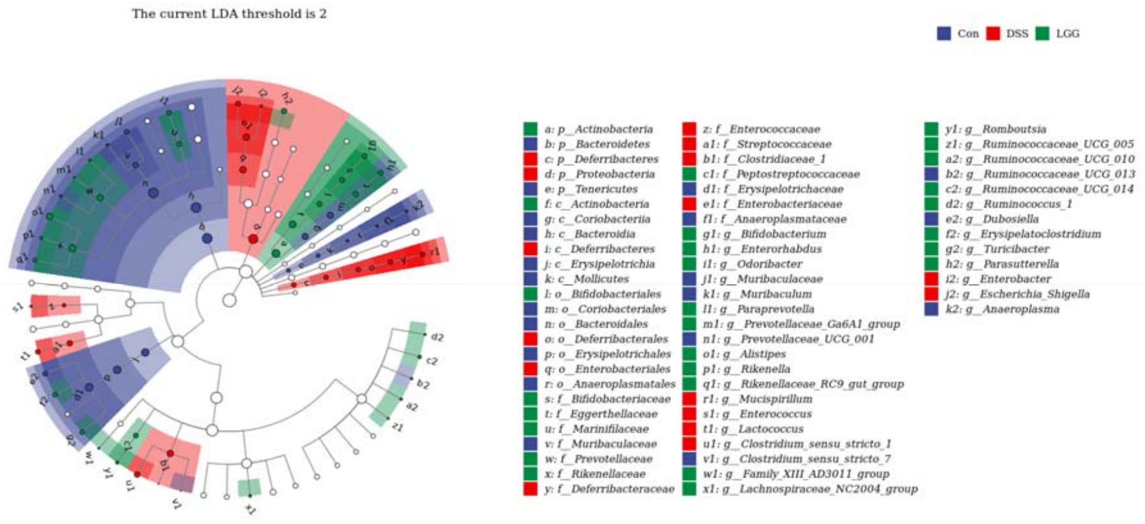

Fig. S2 Bacterial taxa identified as differentially abundant between groups according to linear discriminant analysis effect size (LEfSe).

Table S1 The mice dietary ingredients and nutritional value.

Numbers	Component
1	corn
2	soybean meal
3	flour
4	wheat middlings
5	fish meal
6	plant oil
7	dicalcium phosphate
8	limestone
9	salt
10	vitamins
11	mineral elements
**The energy contribution**	**Nutritional value (%)**
Protein	23.07%
Fat	11.85%
Carbohydrate	65.08%
Total energy	3.40kcal/g
**Vitamin**	**Nutritional value (/kg)**
Vitamin A (IU)	7800.00
Vitamin D(IU)	1200.00
Vitamin E (mg)	67.00
Vitamin K (mg)	5.00
Vitamin B1, 2, 6, 12 (mg)	35.02
Nicotinic acid (mg)	55.00
Biotin (mg)	0.20
Choline (mg)	1250.00
Folic acid (mg)	6.60
Pantothenic acid (mg)	22.00
Amino acid (g)	83.90
Mineral (g)	13.64

Mouse feed was purchased BEIJING KEAO XIELI FEED CO., LTD. More detail information was supported by http://en.keaoxieli.com/product/144.html.

Table S2 Primer sequences for qRT-PCR analysis.

Gene	Forward Primer Sequence	Reverse Primer Sequence
TNF-α	GCGACGTGGAACTGGCAGAAG	GCCACAAGCAGGAATGAGAAGAGG
IL-1β	TCGCAGCAGCACATCAACAAGAG	TGCTCATGTCCTCATCCTGGAAGG
IL-2	GCAGCTCGCATCCTGTGTCAC	CTGCTGTGCTTCCGCTGTAGAG
IL-6	ACTTCCATCCAGTTGCCTTCTTGG	TTAAGCCTCCGACTTGTGAAGTGG
TLR-4	ACAAGGCATGGCATGGCTTACAC	TGTCTCCACAGCCACCAGATTCTC
MyD88	GCTAGAGCTGCTGGCCTTGTTAG	TCTCGGACTCCTGGTTCTGCTG
GAPDH	GGTTGTCTCCTGCGACTTCA	TGGTCCAGGGTTTCTTACTCC

Table S3 Protein levels of IL-1β, IL-6, and TNF-α in serum were determined by ELISA. (n=3)

Groups	TNF-α (pg/mL)	IL-6 (pg/mL)	IL-β (pg/mL)
Control	67.67±1.68**	19.07 ± 1.36*	18.23±0.78**
DSS	71.71±2.05	22.05 ± 2.16	20.43±1.96
DSS+L-EVs	60.52±2.74**	20.29±1.28	18.96±1.41

*$p < 0.05$, **$p < 0.01$ *vs.* DSS group.

Normality of distribution of Data:

For Table S1: The verification of normal distribution.

	-6d	-4d	-2d	0d	2d	4d	6d	8d
D'Agostino & Pearson test								
K2	2.393	0.0881	1.380	0.6798	5.820	0.4083	1.087	5.393
P value	0.3023	0.956	0.5016	0.7118	0.0545	0.8153	0.5808	0.0675
Passed normality test (alpha=0.05)?	Yes	Yes	Yes	Yes	Yes	Yes	Yes	Yes
P value summary	ns	ns	ns	ns	ns	ns	ns	ns

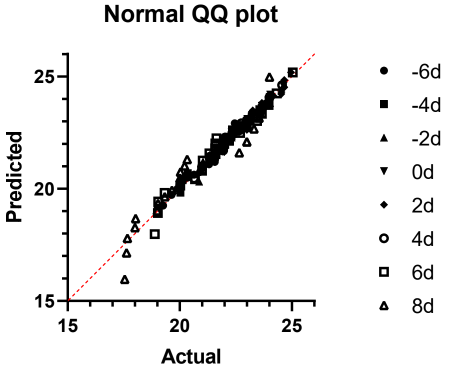

For Table S2: The verification of normal distribution.

	spleen index	liver index	colon length
D'Agostino & Pearson test			
K2	1.017	0.1099	0.2163
P value	0.6013	0.9465	0.8975
Passed normality test (alpha=0.05)?	Yes	Yes	Yes
P value summary	ns	ns	ns

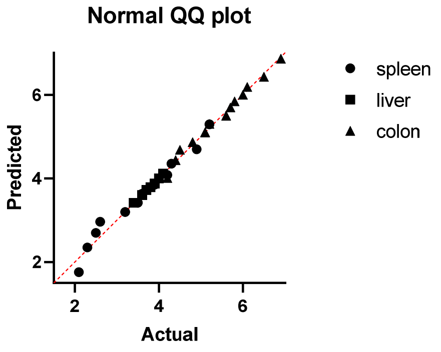

For Table S3: The verification of normal distribution.

	IL6	IL-1b	IL-2	TNF-a	TLR4	Myd88
D'Agostino & Pearson test						
K2	0.5954	2.960	3.136	2.782	3.692	0.5582
P value	0.7425	0.2277	0.2085	0.2488	0.1579	0.7565
Passed normality test (alpha=0.05)?	Yes	Yes	Yes	Yes	Yes	Yes
P value summary	ns	ns	ns	ns	ns	ns

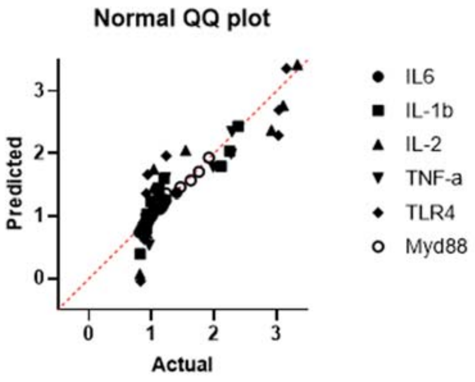

For Table S4: The verification of normal distribution.

	p-p65/p65	ASC	NLRP3
D'Agostino & Pearson test			
K2	1.739	2.963	0.4121
P value	0.4191	0.2273	0.8138
Passed normality test (alpha=0.05)?	Yes	Yes	Yes
P value summary	ns	ns	ns

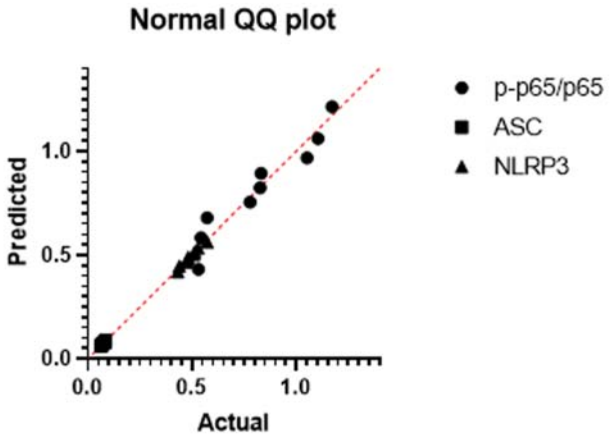

For Table S5: The verification of normal distribution.

	Chao1	Shannon	Observed_species	Faith_pd	Simpson	Pielou_e
D'Agostino & Pearson test						
K2	1.649	0.7036	1.709	0.3403	4.437	2.216
P value	0.4385	0.7034	0.4255	0.8436	0.1088	0.3302
Passed normality test (alpha=0.05)?	Yes	Yes	Yes	Yes	Yes	Yes
P value summary	ns	ns	ns	ns	ns	ns

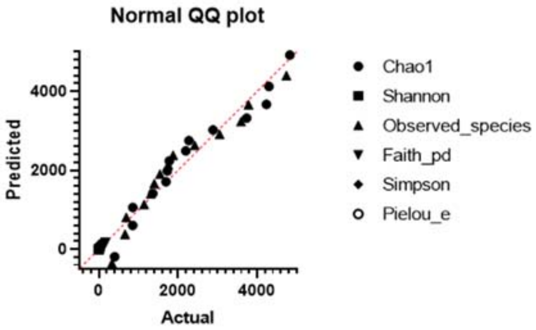

For Table S6: The verification of normal distribution.

	Lachnospiraceae	Ruminococcaceae	Lactobacillaceae	Clostridiaceae_1	Clostridiales_ vadinBB60_group
D'Agostino & Pearson test					
K2	3.836	2.915	8.976	3.244	19.86
P value	0.1469	0.2328	0.0112	0.1975	<0.0001
Passed normality test (alpha=0.05)?	Yes	Yes	No	Yes	No
P value summary	ns	ns	*	ns	****

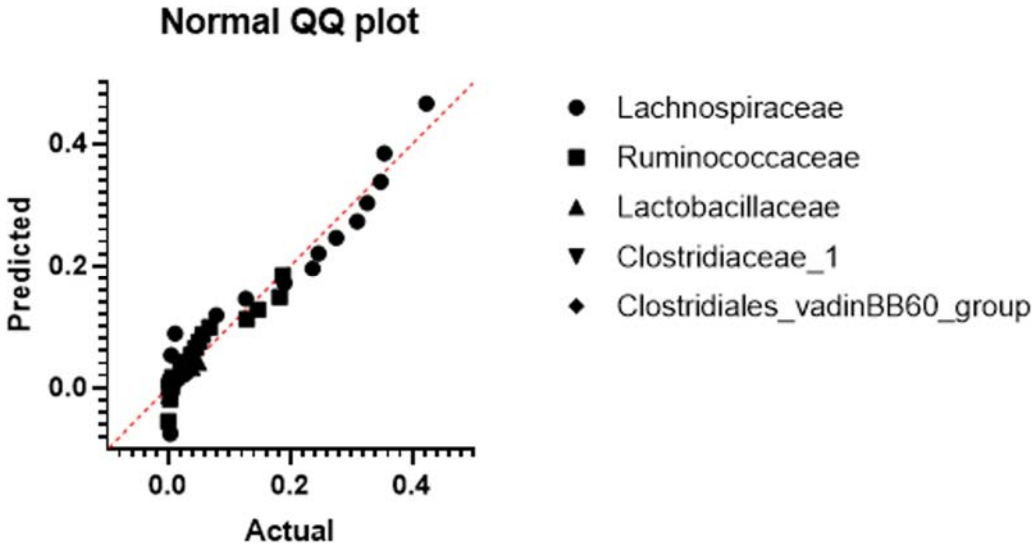

For Table S7: The verification of normal distribution.

	Lachnospiraceae _NK4A136_group	Rumini clostridium_9	Lacto bacillus	Clostridium _sensu_stricto_1	Clostridiales _vadinBB60_group	Faecali baculum
D'Agostino & Pearson test						
K2	4.114	29.91	4.522	3.139	17.73	11.18
P value	0.1278	<0.0001	0.1042	0.2081	0.0001	0.0037
Passed normality test (alpha=0.05)?	Yes	No	Yes	Yes	No	No
P value summary	ns	****	ns	ns	***	**

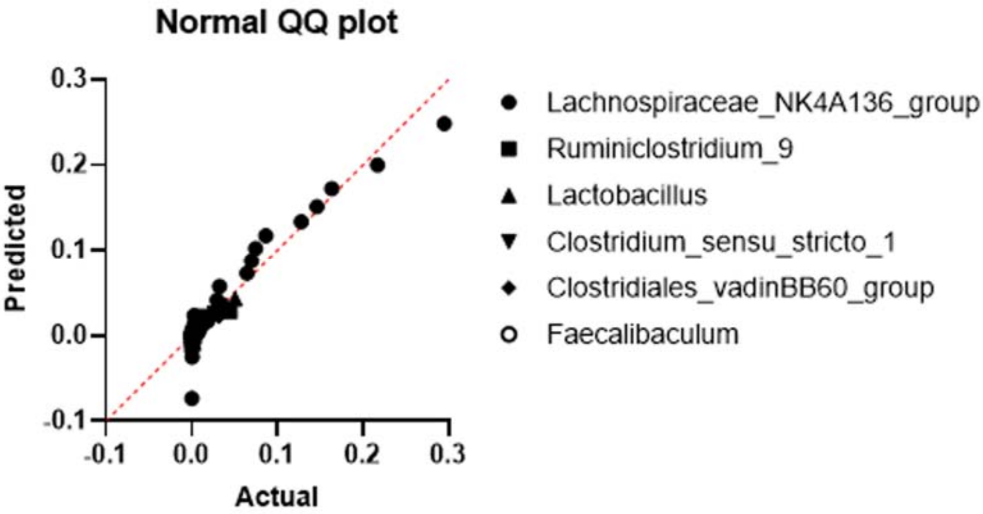

Supplement: Supplementary file 1 [file nutrients-13-03319-s001.zip › nutrients-1381776-supplementary.pdf]
